# Knowledge and use of emergency contraceptives amid women seeking termination of pregnancy in the North West province

**DOI:** 10.4102/phcfm.v17i1.4777

**Published:** 2025-10-25

**Authors:** Elsje van Niekerk, Deidré Pretorius

**Affiliations:** 1 Department of Family Medicine and Primary Care, School of Clinical Medicine, Faculty of Health Sciences, University of the Witwatersrand, Johannesburg, South Africa

**Keywords:** emergency contraceptives, termination of pregnancy, unintended pregnancy, life orientation, unplanned pregnancy, knowledge of emergency contraceptives

## Abstract

**Background:**

Despite acceptable contraceptive coverage rates in South Africa, the rise in the number of termination of pregnancies is worrisome and suggests that family planning services are not yet optimal. Emergency contraceptives are underutilised in South Africa.

**Aim:**

To assess the knowledge and use of emergency contraceptives among women presenting to a termination of pregnancy (TOP) facility.

**Setting:**

The study was conducted in the JB Marks sub-district, North West province, South Africa.

**Methods:**

This cross-sectional study was based at the TOP clinic at Potchefstroom Hospital, North West province. One hundred and ninety-six women completed self-administered questionnaires. Completion of the questionnaire was considered consent. Descriptive statistics were performed, and Chi^2^ and Fischer exact test were used to determine relationships between variables.

**Results:**

The mean age of participants was 26.5 years (standard deviation [s.d.] = 5.87), with 114 (58.2%) participants being single women in their 20s. Among 162 participants who had never used emergency contraceptives, 64.8% cited a lack of knowledge as the primary barrier to use. Only 34 (17.4%) of participants have previously used emergency contraceptives. The main reason for poor uptake among the women who never used emergency contraceptives could be attributed to poor knowledge.

**Conclusion:**

This study highlighted that knowledge and usage of emergency contraception are low in women presenting for TOP in the health sub-district. Emergency contraceptives can reduce the number of unintended pregnancies and its associated trauma significantly. Community intervention should be of utmost importance to improve the knowledge and usage of emergency contraception.

**Contribution:**

This study emphasised the need to make young adults aware of emergency contraceptives to avoid the trauma of unintended pregnancy for women.

## Introduction

Reproductive health policies and laws in South Africa are among the most progressive laws worldwide since the end of the apartheid era.^1^ Contraception use shifted from an agenda of population control to that of empowering men and women in their reproductive decision-making.^1^ In February 1997, the *Choice on Termination of Pregnancy (TOP) Act* was gazetted pertaining to women’s choice of terminating a pregnancy up to 12 weeks of gestation (first trimester); up to 20 weeks of gestation for certain criteria and after 20 weeks only in selective circumstances.^2,3,4^ According to the District Health Information System Database (DHIS), the 2019 national and North West province couple year protection rate (women of childbearing age protected against pregnancy by any contraceptive method) was 54.5% and 62.4%, respectively.^5^ Nationally, the number of reported terminations of pregnancies increased by 48.7% from 2015 to 2019 with 83 707 terminations in 2015 and 124 446 terminations in 2019.^5^ Despite access to contraceptives, including emergency contraceptives and an acceptable contraceptive coverage rate, the rise in the number of terminations of pregnancies suggests that family planning services are not yet optimal.^6^ The psychological trauma associated with the termination of pregnancies could be avoided with the adequate use of contraceptives and emergency contraceptives.

As much as 66% of all pregnancies in South Africa are unintended.^7^ The limited knowledge of and misconceptions about the use of contraceptives, including that of emergency contraception, are worrisome.^6,8,9^ According to the World Health Organization (WHO), an estimated 308 million unintended pregnancies were prevented by modern contraception during 2017.^10^ Despite the availability of modern contraception, unintended pregnancies remain high and lead to increased maternal mortality rates. Sub-Saharan Africa contributes more than 60% of the total maternal deaths globally and 75% of abortions performed in Africa are unsafe, with a high mortality rate.^11^ This highlights the importance of decreasing the number of unintended pregnancies with the use of contraceptives, including emergency contraceptives, as a key factor to decrease maternal mortality in line with the approach to monitoring health for the United Nations Sustainable Development Goals.^11^

Emergency contraceptives, a contraceptive method that can be used to prevent pregnancy in the event of method failure, unprotected intercourse or the incorrect use of an existing contraceptive method,^8^ have become available at primary health care clinics and at all pharmacies in the private sector in South Africa without prescription since 2000.^1,8,12^ South Africa is one of a few countries that offer this service without a doctor’s prescription in order to improve accessibility.^8^ Emergency contraceptives, if taken within 72 h to 120 h, can reduce the risk of an unwanted pregnancy by 75% – 99%.^9,12^

The current South African literature on the knowledge and use of emergency contraceptives, among women, including women presenting for a TOP, shows that this population has poor knowledge and low use of emergency contraceptives.^9,12,13,14,15,16,17,19,20,21^ Most of these studies were performed in the KwaZulu-Natal, Gauteng and Western Cape provinces and included urban and rural areas. The knowledge and use of emergency contraceptives was lower among rural public healthcare users,^12,17,18^ and lower awareness was seen in women with a lower level of education.^12,13,20^ There seems to be a discrepancy between the knowledge and use of emergency contraceptives among women presenting for TOP in KwaZulu-Natal province, where knowledge around the use of emergency contraceptives was adequate, but very low uptake of emergency contraceptives was reported.^13^ There is currently no data available on the knowledge and use of emergency contraceptives among women presenting for TOP in the North West province, South Africa.

Therefore, this study aimed to assess the knowledge and use of emergency contraceptives among women presenting to a TOP facility in the JB Marks sub-district, North West province, South Africa.

## Research methods and design

### Study design

This was a cross-sectional analytical study design. It was an appropriate design as it reflects a moment in time for participants who do not frequent the facility routinely.

### Setting

The study was based at Potchefstroom Hospital’s TOP clinic, Potchefstroom, South Africa. This is an urban-based regional hospital serving the community of JB Marks sub-district and performs about 500 TOPs annually.^22^ Although the clinic is in an urban setting, JB Marks sub-district includes multiple peri-urban and rural areas with a population of 184 835 (48% female).^23^ It is also the site for a university and various colleges and thus, at times, a high congregation of young adults.

### Study population and sampling strategy

A minimum sample size of 235 was calculated with a 5% margin of error and a 95% confidence interval.^24^ During the coronavirus disease 2019 (COVID-19) pandemic, a decline of 29% in TOP requests was noted, and the sample size was adjusted to 205. All the adult women (> 18 years) were recruited in a consecutive manner as they presented to the clinic requesting a first-trimester TOP. A woman who appeared physically or emotionally unwell would have been excluded from the study and referred to an appropriate healthcare provider.

### Data collection tool

The self-administered questionnaire was based on the questionnaire used in a 2014 study conducted in the KwaZulu-Natal province that investigated the self-reported knowledge and use of emergency contraceptives among women presenting for TOP,^13^ after permission was obtained from the author. The questionnaire consisted of three sections: socio-demographic questions, knowledge of emergency contraceptives and usage of emergency contraceptives. The data were captured by the principal investigator on a password-protected computer using Excel, 2010 version (Microsoft, United States [US]).

### Data collection

Participants were provided with a study information sheet and a sealed self-administered questionnaire. If the participant consented to participate, she then completed the anonymous questionnaire in a private room, sealed the completed questionnaire in an envelope and placed it in a sealed box. If the participant wished not to participate, the incomplete questionnaire was placed in a sealed envelope and put in the same sealed box. The principal investigator and research assistant were available outside the room for any questions or queries, such as difficulty understanding the questions or being not read or write. Data were collected from November 2020 to July 2021. The local and national COVID-19 precautionary guidelines were followed throughout this process. The planned data collection period of 4 months was extended by another 5 months to reach the sample size within the limitations set by the COVID pandemic.

### Data analysis

The data were analysed using SAS (SAS Institute Inc., Carey NC, US), Release 9.4. Descriptive statistics were used to describe the findings for all the variables. The Chi-square test was used to look at associations between area of residence and emergency contraceptive knowledge. The Fisher exact test was used (small sample) to look at associations between level of education and knowledge of emergency contraceptives. Knowledge scores were calculated to assess the level of knowledge about emergency contraceptives. These scores were based on correctly answering the questions about emergency contraceptives and were interpreted as follows: 0% – 40% was considered poor knowledge, 41% – 60% moderate knowledge and 61% – 100% good knowledge.

### Ethical considerations

Ethical clearance to conduct this study was obtained from the Human Research Ethics Committee, University of the Witwatersrand (No. M200575), North West Department of Health and Potchefstroom Hospital (RS31/02/2020). Informed consent forms were not used as they would contain personal identifiers of this vulnerable population, and verbal consent and willingness to participate were deemed sufficient. The research method provided full privacy and confidentiality. As no person assisted with the completion of forms or received the forms for immediate analysis, it is not possible to identify distress during completion of the questionnaires. Therefore, the study information sheet contained details on how to access counselling if the participants required extra support.

## Results

During the 9-month data collection period, 390 women were scheduled to do a TOP at the study site, and all were recruited to participate. Two hundred and five took the questionnaires to complete. Nine incomplete questionnaires were excluded as this was considered non-consent. The mean age of participants was 26.5 years (standard deviation [s.d.] = 5.87), with 114 (58.2%) participants being single women in their 20s. The socio-demographic profile of the participants is presented in Table 1. Among 162 participants who had never used emergency contraceptives, 64.8% cited a lack of knowledge as the primary barrier to use (Table 2). Only 34 (17.4%) participants have previously used emergency contraceptives, and the emergency contraceptive of choice was oral contraceptive pills (100%), and 32 (94.1%) participants obtained it from a private pharmacy. The main reason for poor uptake among the women who never used emergency contraceptives could be attributed to poor knowledge (see Table 2).

**TABLE 1:** Socio-demographic information (*N* = 196).

Variable	Mean	s.d.	Median	IQR	Minimum	Maximum	*n*	%
**Age (years)**	26.5	5.87	25	21–31	18	45	-	-
**Relationship status (*n* = 196)**								
Divorced	-	-	-	-	-	-	4	2.0
Married	-	-	-	-	-	-	10	5.1
Single	-	-	-	-	-	-	114	58.2
Engaged or promised	-	-	-	-	-	-	2	1.0
Separated	-	-	-	-	-	-	3	1.5
In a relationship	-	-	-	-	-	-	63	32.2
**Highest level of education completed (*n* = 196)**								
Grade 1 – Grade 7	-	-	-	-	-	-	3	1.5
Grade 8 – Grade 11	-	-	-	-	-	-	38	19.4
Grade 12	-	-	-	-	-	-	124	63.3
Diploma	-	-	-	-	-	-	14	7.1
Tertiary education	-	-	-	-	-	-	17	8.7
**Current university student (*n* = 196)**								
Yes	-	-	-	-	-	-	43	21.9
No	-	-	-	-	-	-	153	78.1
**Occupation (*n* = 196)**								
Employed	-	-	-	-	-	-	57	29.1
Part time or piece job	-	-	-	-	-	-	28	14.3
Self-employed	-	-	-	-	-	-	4	2.0
Student	-	-	-	-	-	-	56	28.6
Unemployed	-	-	-	-	-	-	51	26.0
**Residence (*n* = 196)**								
Formal housing town	-	-	-	-	-	-	65	33.1
Formal housing in township	-	-	-	-	-	-	86	43.9
Informal housing in town	-	-	-	-	-	-	16	8.2
Informal housing in township	-	-	-	-	-	-	27	13.8
On a farm	-	-	-	-	-	-	2	1.0
**Who do you live with (*n* = 196)**								
Alone	-	-	-	-	-	-	15	7.7
Friends	-	-	-	-	-	-	9	4.6
Family	-	-	-	-	-	-	149	76.0
Communal living	-	-	-	-	-	-	23	11.7
**How many children do you have (*n* = 196)**								
0	-	-	-	-	-	-	68	34.7
1–2	-	-	-	-	-	-	104	53.1
3–4	-	-	-	-	-	-	24	12.2
**Number of abortions (*n* = 196)**								
First	-	-	-	-	-	-	161	82.1
Second	-	-	-	-	-	-	26	13.3
Third	-	-	-	-	-	-	9	4.6

s.d., standard deviation; IQR, interquartile range.

**TABLE 2:** Knowledge and use of emergency contraceptives (*N* = 196).

Knowledge and use	*n*	%	95% CI
**Knowledge (*n* = 196)**			
Poor	108	55.0	48.1% – 61.9%
Moderate	54	27.6	21.8% – 34.2%
Good	3	17.4	12.7% – 23.3%
**Have you used emergency contraception (*n* = 196)**			
Yes	34	17.4	-
No	162	82.6	-
**Emergency contraceptive used (*n* = 34)**			
Pills	34	100.0	-
**Where did you get emergency contraceptive (*n* = 34)**			
Government hospital	2	5.9	-
Private pharmacy	32	94.1	-
**Why have you never used emergency contraceptives (*n* = 162)**			
Did not have money to buy it	14	8.6	-
Did not know about it	105	64.8	-
Did not think about it	15	9.3	-
Did not think I would get pregnant	13	8.0	-
Did not know where to get it	4	2.5	-
It was too late to use it	11	6.8	-

CI, confidence interval.

There was no observed relationship between area of residence and emergency contraceptive knowledge (*p* = 0.129). Furthermore, there was no statistical relation between level of education (Grade 12 and higher) and emergency contraceptive knowledge (*p* = 0.197). A logistic regression analysis was performed with use of emergency contraception (yes or no) as dependent variable and knowledge (good or moderate or poor) as predictor variable, modelling the probability that emergency contraception is used (Yes). Knowledge was found to be a significant predictor (*p* < 0.001). Calculating an odds ratio for good knowledge versus poor knowledge, it was 86 times more likely to use emergency contraception when the person had good knowledge compared to poor knowledge. Similarly, having moderate knowledge suggested 14 times more likely use of emergency contraception compared to having poor knowledge.

Using a 95% confidence interval, a range was calculated for knowledge to assess that the data were reliable (see Table 2).

## Discussion

The second leading cause contributing to the global burden of disease, is unsafe sex.^25^ Therefore, this study aimed to assess the knowledge and use of emergency contraceptives among women presenting to a TOP facility in the JB Marks sub-district, North West province. The main findings of this study included the decline in TOP requests, the relationship status of the women, their knowledge about emergency contraceptives and their reasons for the low uptake of emergency contraceptives.

Data collection was hampered by the COVID-19 pandemic that caused a decline in TOP requests. For the period 2020 and 2021, a 29% reduction in termination of pregnancies was documented compared to 2019 and 2020.^26^ This was similar to other countries.^27,28,29,30,31^ A general decline in termination of pregnancies was also reported during 2020 in the Gauteng province,^32^ and according to the 2020 DHIS health indicators, there was a national decline of 17%.^5^ Reasons for the decline are still unclear, but it is thought that access to reproductive healthcare and fear of contracting the coronavirus were contributing factors to the low numbers of TOP requests.^27,28,29,31^ Schooling was also online and thus low numbers of students on tertiary campuses. Furthermore, the influence and opinions of partners, spouses or family members who lived in COVID isolation with these women as potential users of the TOP clinic must not be underestimated as a factor preventing them from presenting for a TOP.^32^ The United Nations predicted 116m unintended pregnancies because of poor access to contraceptives during the COVID-19 lockdowns^33^ and up to 2.7m additional unsafe abortions that could have been performed as a consequence of the pandemic.^34^ Even though TOP was deemed an essential service during the COVID-19 pandemic in South Africa with no hindrance in access, less women presented for TOP services.

The socio-demographic profile of this study sample was similar to that of the previous study from the KwaZulu-Natal province.^13^ Most of the participants presenting for a TOP were young single women. Unintended pregnancies not only affect the individual but also their families and extended families. The majority of the women in this study reported that they lived with family, which is in line with other South African studies.^35,36^ Their being single, however, does not mean that their sexual partner or family did not play a role in their decisions to terminate the pregnancy. Unintended pregnancies can destabilise their support structure, increase conflict with family and lead to increased social and financial stress to both the family and individual.^37^

Not only will socio-economic factors influence the family, but there can also be increased levels of stress and conflict with the sexual partner.^37^ More than a third of the study participants reported that they were either in a relationship, engaged or married. This leads to the speculation about the role that the partners played in the decision to prevent or terminate the pregnancy, as well as the support that was offered to the women post-termination. The choice to terminate the pregnancy is often only the tip of the iceberg of the social and emotional stressors that women with unintended pregnancies experience. Circumstances such as intimate partner violence, gender inequality and access to contraception can influence their ability to prevent unintended pregnancies.^37,38,39,40^ Keeping this in mind, disclosure of an unintended pregnancy to a sexual partner and the decision to terminate the pregnancy might have negative repercussions in terms of safety, support, finances and resources. Whether it is a determinant of the choice of termination or a possible consequence of a termination, it can be to the woman’s detriment. This highlights the importance to assess for social safety and to screen for intimate partner violence during the TOP counselling session. Unfortunately, in the *Choice on Termination of Pregnancy Act*, counselling for intimate partner violence is advised, but it is not mandatory.^3^ Previous studies have highlighted that some women experience coercion in terms of sexual and reproductive decision-making and that their autonomy often took a back seat.^32^ Their choice to prevent or terminate the pregnancy might not even be their own choice in such circumstances. It is thus reasonable to conclude that using contraceptives, including emergency contraceptives, may empower women to have more autonomy over their own reproductive choices and increase their safety and stability in partner and family relationships.

Most of the participants had a poor level of knowledge regarding emergency contraceptives (55%), and this result was in keeping with previous South African studies.^9,12,14,15,16,17,19,20,21^ Contrary to these studies,^12,13,17,18,20^ the current study sample did not show any significant relationship between the level of education or area of residence. However, the level of knowledge regarding emergency contraceptives and the likelihood of use were statistically associated. This emphasised the value of knowledge. Comprehensive sexuality education (CSE) has been part of the South African Life Orientation (LO) school curriculum since 2000, where emphasis is placed on safe sexual practices, including contraception.^38^ Emergency contraception is covered extensively in the Grade 9 LO workbook. This workbook includes a good description of emergency contraceptives, how it works, where to obtain it and when to use it.^41^ The majority of women in this study (79%) had at least a Grade 12 qualification and were likely to have completed CSE in LO at school. This issue is therefore much more complex and deeper than purely educating women on safe sexual practices, contraception and emergency contraception use. It has been reported that LO educators do not teach anything about contraception except the failure rates and enforce an abstinence agenda.^42,43^ This implies that they never learn that emergency contraceptives are intended for occasional use rather than routine family planning, either. The educators tend to advocate their personal morals, culture and ideas instead of teaching the outcomes as instructed by the South African Department of Basic Education.^42,43^ Life orientation is not prioritised by schools or learners as it is not considered for tertiary education admission.^44^ Educators also feel that they are not qualified to teach LO, and it is believed that anyone can teach LO even if they lack knowledge regarding the content.^44^ Thus in theory, we believe that South African teenagers receive CSE, but in reality, we have to question the delivery of this education. Unintended pregnancies are on the rise in South Africa^45^ and the LO curriculum should be prioritised in schools. Teenagers should be health literate and equipped to make educated decisions regarding their reproductive health and emergency contraceptives if they do not want to use the longer-acting contraceptives.

The uptake of emergency contraception was low, with 17.4 % of the participants in this study reporting that they had used emergency contraceptives. It was reported that they lacked mainly knowledge of emergency contraception. Unfortunately, the poor knowledge also contributed to the poor uptake of emergency contraceptives. Of concern was the 8% who did not think that they would get pregnant, and it would have been interesting to determine whether they used other modern or cultural methods of birth control or just did not understand the biology of intercourse and pregnancy.^46,47,48^

Unintended pregnancies have complex and interconnected roots that need to be explored to provide comprehensive care to the woman requesting a TOP. Reasons for TOPs in South Africa are often multi-factorial and mainly socio-economical in nature.^35,36^ This study highlighted that knowledge and usage of emergency contraception is low in women presenting for TOP in the JB Marks sub-district, despite school programmes on reproductive health.

### Limitations and bias

Recruiting all the women presenting for TOP, there is a limited selection bias, which is also a strength of this study. Data were collected during the COVID pandemic, and results must be interpreted within the limitations the pandemic set on patients’ access to TOP facilities and contraceptives. Knowledge of emergency contraceptives was, however, independent of the pandemic. The questionnaire had only four items measuring knowledge of emergency contraception and could be considered a limitation; however, for the purpose of a dichotomous variable, it was considered sufficient for this research. It was not possible to consider language and literacy competencies before participants were recruited, as confidentiality was prioritised. This could be a limitation that some women did not feel comfortable completing the questionnaires and thus did not have the opportunity to add to this study.

### Recommendations

This study alerted us that primary care facilities must advocate for more information and support to women to access emergency contraceptives at primary care level and not hospital dispensaries or private pharmacies. Increasing awareness and accessibility of a range of contraceptive options, along with proper guidance on emergency contraception for occasional use, could improve family planning outcomes. Community and primary health care interventions should aim to improve not only the knowledge and usage of emergency contraception but also provide women with a safe platform to report any injustice that led to the unintended pregnancy. It is also recommended that this study be repeated in the same setting in nonpandemic circumstances.

## Conclusion

The implications of an unintended pregnancy are multifactorial and complex. If the choice is made to continue with pregnancy, it might be associated with poor antenatal care, increased maternal and child mortality and psychosocial stress, to name a few. If the choice is made to terminate, there might be short-term or long-term physical, emotional and social trauma sequelae. Emergency contraceptives can reduce the number of unintended pregnancies and its associated trauma significantly and should be utilised to its full potential. This study highlighted the importance of improving emergency contraceptive knowledge in schools, universities and primary health care.
